# Circulating *miR-34a* and *miR-125b* as Promising non Invasive Biomarkers in Egyptian Locally Advanced Breast Cancer Patients 

**DOI:** 10.31557/APJCP.2019.20.9.2749

**Published:** 2019

**Authors:** Neemat M Kassem, Wael S Makar, Hebatallah A Kassem, Soha Talima, Mustafa Tarek, Hadeer Hesham, Mohamed A El-Desouky

**Affiliations:** 1 *Department of Clinical and Chemical Pathology, *; 2 *Department of Clinical Oncology,*; 3 *Molecular oncology Unit, Kasr Al Ainy Centre of Clinical Oncology and Nuclear Medicine, School of Medicine,*; 4 *Faculaty of Science, Cairo University, Cairo, Egypt. *

**Keywords:** Breast cancer, circulating microRNA, biomarker, neoadjuvant chemotherapy

## Abstract

**Background:**

Breast cancer (BC) is the second most common cancer worldwide. MicroRNAs are a group of non-coding, single stranded RNAs of ~ 22 nucleotides, which regulate gene expression at the post-transcriptional level. Circulating miRNAs have been found as potential blood based predictive biomarkers.

**Purpose:**

we aim to evaluate *miR-34a *and *miR-125b* to predict outcome from neoadjuvant chemotherapy in Egyptian BC patients.

**Methodology:**

Quantitative assessment of plasma *miR-34a* and *miR-125b* expression was performed by qRT-PCR. Thirty nine newly diagnosed locally advanced BC female patients with 10 age and sex matched healthy volunteers were included in the study.

**Results:**

We performed ROC curve analysis to evaluate the diagnostic value for the miR-34a with AUCs = 0.995, cutoff point of 2.57 sensitivity 97.4%, specificity 100%, PPV 100%, NPV 83.3% and accuracy 97.7%.*miR-125b* had AUC = 0.68 and a cutoff point of 8.69 with sensitivity 66.7%, specificity 70.0%, PPV 90.6%, NPV 41.2% and accuracy 73.5%. *miR-34a *expression were significantly higher in BC patients compared to controls with p value <0.001*. Also, *miR-34a* expression level was significantly higher in patients with progressive disease with P value =0.03*. However, *miR-125b* expression levels were insignificantly higher in responsive patients with p value = 0.2.

**Conclusion:**

miRNAs are crucial candidates for novel molecular targeted therapies due to their capability to regulate numerous genes in molecular pathways. Our data suggest that circulating miR-34a and miR-125b expression levels could be promising highly accurate non-invasive biomarkers in diagnosing BCs. miR-34a can predict chemotherapeutic resistance associated with higher expression levels in non-responsive patients.

## Introduction

Breast cancer (BC) is the second most common cancer worldwide and is the most common cancer among Egyptian females (Talima et al., 2019). In Egypt, cancer incidence rates at national and regional level was published based upon results of National Cancer Registry Program (NCRP). This registry represented that the commonest sites were liver (23.8%), breast (15.4%), and bladder (6.9%) in both genders, liver (33.6%) & bladder (10.7%) in males, and breast (32.0%) and liver (13.5%) in females (Ibrahim et al., 2014). MicroRNAs (miRNAs) are a group of non-coding, single stranded RNAs of ~ 18-24 nucleotides, which regulate gene expression at the post-transcriptional level (Doench and Sharp, 2004). miRNAs modulate numerous cellular pathways, such as cell proliferation, differentiation and apoptosis and may function as oncogenes or tumor suppressing genes. Circulating miRNAs have been found as potential blood based biomarkers for cancer detection (Yu et al., 2011). Among the oncogenic *miRNAs*, *miR-34a* and* miR-125b* which have been reported to be related to breast cancer. *miR-34* gene which located on lp36.23 is a tumour suppressor gene direct downstream component of the *p53* network . It was found that miR-34a is down-regulated in breast cancer cell lines and tissues, compared with normal cell lines and the adjacent non-tumor tissues, respectively (Li et al., 2012). It was reported that ectopic expression of miR-34a inhibits breast cancer cells growth, invasion and migration. It contributes to drug resistance of breast cancer by targeting many oncogenes. It interacts with *BCL-2* and *CCND1* and is reported to be associated with docetaxel resistance. By targeting NOTCH 1 and protein kinase D1 (PRKD1), *miR-34a* modulates chemosensitivity of breast cancer cells to adriamycin, and stimulates breast cancer stemness and drug resistance, respectively. *miR 125b* is a tumor suppressor in breast tumorigenesis, its overexpression leads to reduced migration and invasion capacities. It can also induce metastasis by targeting StAR related lipid transfer domain containing 13 (STARD13) in MCF 7 and MDA MB 231 breast cancer cells (Li et al., 2017; Tang et al., 2012). Also, upregulation of miR 125b conferred to chemoresistance by targeting B cell lymphoma 2 antagonist killer 1 (Bak1), and it could maintain cancer stem like side population fraction. Finally, it was reported that circulating *miR 125b* expression was associated with chemotherapeutic resistance of breast cancer (Luo et al., 2017). There are many molecular mechanisms that may contribute to chemotherapeutic resistance in breast cancer patients. So, we aim to evaluate 2 potential biomarkers: *miR-34a* and* miR-125b *in diagnosis and to predict outcome from neoadjuvant chemotherapy in locally advanced Egyptian breast cancer females.

## Materials and Methods

*Study population*

The present study included 39 newly diagnosed locally advanced breast cancer female patients. Patients were recruited from the outpatient clinic of Kasr Al-Ainy Center of Clinical Oncology and nuclear medicine, School of Medicine, Cairo University, in the period from October 2017 to February 2018. All the patients have been prepared to receive pre-operative neoadjuvant chemotherapy with an association of anthracycline and taxanes for 8 cycles during 6 months. Ten age- and sex-matched healthy volunteers were included in the study as a control group. The study was approved by the Research ethical Committee of Clinical Oncology department, School of Medicine, Cairo University, and informed consents were obtained from all participants prior to enrollment in the study.

*Cell-free total RNA extraction*

For the patients and controls, 3 ml peripheral blood sample was collected under complete aseptic conditions for molecular studies. 200 µl EDTA plasma was separated and used for extraction of all RNA molecules from approximately 18 nucleotides (nt) upwards by miRNeasy Serum/Plasma kit (Qiagen, Valencia, CA, USA) according to manufacturer’s instructions. The RNA was eluted in 15 µl RNAse free water, the integrity was tested on the Nanodrop (ND-1000) and finally, RNA stored at −80◦C till used in real time PCR reaction. Qiagen offers miScript Serum/Plasma Spike-In Control (a synthetic C. elegans miR-39) which was added to the homogenized plasma samples prior to RNA purification, CT values obtained using the C. elegans miR-39 miScript Primer Assay can be used to calibrate the data sets being analyzed. This calibration can resolve differences in recovery that may occur during the purification procedure and in amplification efficiency. 

*Quantitative assessment of miRNA-34a and miRNA-125b expression*

One hundred ng of total RNA was reverse transcribed using miScript II RT Kit followed by real-time PCR on StepOne Real-Time PCR machine (Applied Biosystems) using an miRNA-specific miScript Primer Assay (forward primer) for {miR16 (reference miR), *miR34a *and *miR125b*} and the miScript SYBR Green PCR Kit, which contains the miScript Universal Primer (reverse primer) and QuantiTect SYBR Green PCR Master Mix as described by the manufacturer. Samples, validated endogenous controls and interassay controls were used throughout. The relative quantification (RQ) of miRNA gene expression was assessed by 2^−ΔΔCt^ method (ΔΔCt = {[Ct (miRNA of interest) – Ct (reference miR-16 of interest)] − [Ct (miRNA of control) – Ct (reference miR-16 of control)]}.

*Data analysis*

Statistical analysis was done using IBM© SPSS© Statistics version 22 (IBM© Corp., Armonk, NY, USA). Numerical data were expressed as mean and standard deviation or median and range as appropriate. Qualitative data were expressed as frequency and percentage. For not normally distributed quantitative data, comparison between two groups was done using Mann-Whitney test (non-parametric t-test). Comparison between 3 groups was done using Kruskal-Wallis test (non-parametric ANOVA). Spearman-rho method was used to test correlation between numerical variables. The Receiver Operating Characteristic (ROC) curve was used for prediction of cut off values. Evaluation of diagnostic value of the miRNAs was done by calculating sensitivity, specificity, positive predictive values (PPV), negative predictive value (NPV) and accuracy. All tests were two-tailed. A p-value < 0.05 was considered significant.

## Results

*Patients’ characteristics*

Thirty nine newly diagnosed locally advanced breast cancer female patients were included in the study. Their age ranged between 29 and 66 years with mean ± SD = 45.4 ± 9 years and median of 45 years. As regards menopausal status, 23/39 (59%) patients were pre-menopausal and 16/39 (41%) patients were post-menopausal. Regarding body mass index (BMI), 7/39 (17.9%) patients were normal, 21/39 (53.8%) patients were obese and 11/39 (28.2%) patients were overweight. Seventeen (43.6%) patients had tumor on left side, 21/39 (53.8%) had tumor on right side and only one patient (2.6%) had tumor on both sides. Regarding, tumor histology, 37/39 (94.9%) patients had invasive ductal carcinoma (IDC), only one patient had invasive lobular carcinoma (ILC) and one had mixed type. Majority of patients were grade II: 36/39 (92.3%) and only 3/39 (7.7%) were grade III. Regarding TNM stage, 9/39 (23.1%) patients were T2, 13/39 (33.3%) patients were T3 and17/39 (43.6%) patients were T4. Nine patients (23.1%) were N0, 23/39 (59%) patients were N1, 2/39 (5.1%) patients were N2 and 5/39 (12.8%) patients were N3. All patients are non metastatic (M0). Regarding hormone receptor status, estrogen receptor (ER) levels were positive in 29/39 (74.4%) patients and negative in 10/39 (25.6%) patients. Progesterone receptor (PR) levels were positive in 32/39 (82.1%) patients and negative in 7/39 (17.9%) patients. Human epidermal growth factor receptor 2 (HER2/neu) levels were positive in 14/39 (35.9%) patients and negative in 25/39 (64.1%) patients. KI67 were high in 31/39 (79.5%) patients, low in 7/39 (17.9%) patients and missing result in only 1/39 (2.6%) patient. As regards Molecular subtypes, 5/39 (12.8%) patients were luminal A, 29/39 (74.4%) patients were Luminal B, 2/39 (5.1%) patients were Her2 positive disease and 3/39 (7.7%) patients were Triple negative disease.

**Table 1:** Patients` Characteristics

Patients` characteristics	No. (%)
Age (years)	
Range	29-66
Mean ± SD	45.4 ± 9
Median	45
Comorbidities	
DM	1/39 (2.6%)
HTN	3/39 (7.7%)
Both	5/39 (12.8%)
No	30/39 (76.9%)
Body mass index (BMI)	
Normal	7/39 (18%)
Obese	21/39 (53.8%)
Overweight	11/39 (28.2%)
Menopausal status	
Pre	23/39 (59%)
Post	16/39 (41%)
Tumor side	
Left	17/39 (43.6%)
Right	21/39 (53.8%)
Both	1 (2.6 %)
Multicentric tumor	
Yes	8 (20.5%)
No	31 (79.5%)
Tumor histology	
IDC	37/39 (94.9%)
ILC	1/39 (2.6%)
Mixed	1/39 (2.6%)
Grade	
II	36/39 (92.3%)
III	3/39 (7.7%)
TNM stage	
Tumor size	
T2	9/39 (23.1%)
T3	13/39 (33.3%)
T4	17/39 (43.6%)
Lymph nodes	
N0	9/39 (23.1%)
N1	23/39 (59%)
N2	2/39 (5.1%)
N3	5/39 (12.8%)
Metastasis	
M0	39/39 (100%)
Immunohistochemistry	
ER	
Positive	29/39 (74.4%)
Negative	10/39 (25.6%)
PR	
Positive	32/39 (82.1%)
Negative	7/39 (17.9%)
HER2neu	
Positive	14/39 (35.9%)
Negative	25/39 (64.1%)
KI67	
High	31/39 (79.5%)
Low	7/39 (17.9%)
Unknown	1/39 (2.6%)
Molecular subtypes	
luminal A	5/39 (12.8%)
luminal B	29/39 (74.4%)
Her2 positive disease	2/39 (5.1%)
Triple negative disease	3/39 (7.7%)

DM, Diabetes mellitus; HTN, Hypertension; ER, Estrogen receptor; PR, Progesterone receptor; HER2, human epidermal growth factor receptor 2; IDC, invasive ductal carcinoma; ILC, invasive lobular carcinoma

**Table 2:** miR-34a Expression in Breast Cancer Patients

Items	Subgroups	No.	Mean ± SD	Median	Range	P-value
miR-34a expression	BC patients	39	1643.4 ± 3813.6	47.3	1.54 - 17020.7	<0.001*
	Controls	10	1.13 ± 0.54	1.13	0.52 -1.95	
Menopausal status	Pre	23	2232.7±4342.2	75.32	1.54-17020.7	0.12
	Post	16	796.3±2809.5	31.78	3.19-11307.6	
BMI	Normal	7	48.48±80.84	12.3	11.2-229.9	0.17
	Obese	21	1291.8±3001.5	59.1	4.42-11307.6	
	Overweight	11	3329.6±5663.6	75.3	5.08-17020.7	
Multicentric tumor	Yes	8	1245±2751.1	18.7	3.19-7831.1	0.29
	No	31	1746.2±4074.7	63.3	1.54-17020.7	
Grade	II	36	1554.3±3890.4	45.45	1.54-17020.7	0.23
	III	3	2713.1±3098.5	2012.8	24.68-6101.7	
TNM stage:	T2	9	1219.8±2386.1	12.7	1.54-6101.7	0.07
Tumor size	T3	13	3059.9±5491.3	229.9	5.08-17020.7	
	T4	17	784.5±2561.95	37.7	3.19-10550.3	
	N0	9	3971.1±5788.2	95.3	1.54-17020.7	0.79
	N1	23	1217.1±3118.4	63.3	4.42-11307.6	
Lymph nodes	N2/N3	7	51.46±49.04	61.6	12.25-155.96	
Immunohistochemistry:						
ER	Positive	27	1346.3±3214.5	24.68	1.54-11307.6	0.02*
	Negative	12	2505.2±5305.6	192.94	25.9-17020.7	
PR	Positive	30	1064.2±2643.4	31.78	1.54-11307.6	0.01*
	Negative	9	4291.5±6785.0	229.92	69.79-17020.7	
HER2neu	Positive	13	429.7±1232.8	21.43	1.54-4656.4	0.22
	Negative	26	2323.1±4567.7	63.34	3.19-17020.7	
KI67	High	31	2060.3±4188.8	75.32	1.54-17020.7	0.03*
	Low	7	28.4±28.5	12	4.42-69.8	
Molecular subtypes	luminal A	5	24.9±25.2	12	5.08-6.3	0.08
	luminal B	29	1536.6±3249.7	43.6	1.54-11307	
	Her2 positive disease	2	149.9±113.2	149.86	69.8-229.9	
	Triple negative disease	3	6369.6±9274.8	2012.82	75.3-17020.7	

*, Significant at P ≤ 0.05

**Table 3:** miR-125b Expression in Breast Cancer Patients

Items	Subgroups	No.	Mean ± SD	Median	Range	P-value
miR-125b expression	BC patients	39	21.7±52.1	4.75	0.28-260.8	0.2
	Controls	10	1.04±0.35	0.92	0.7-1.53	
Menopausal status	Pre	23	24.7±55.3	6.2	0.4-260.8	0.05*
	Post	16	17.3±48.5	1.96	0.28-194.96	
BMI	Normal	7	6.7±7.1	4.9	0.42-20.9	0.4
	Obese	21	16.2±42.2	2.7	0.28-194.9	
	Overweight	11	41.7±77.8	10.8	0.4-260.8	
Multicentric tumor	Yes	8	9.7±13.7	3.04	0.43-40.7	0.9
	No	31	24.75±57.85	4.9	0.28-260.8	
Grade	II	36	20.7±52.7	4.8	0.28-260.8	0.9
	III	3	32.8±52.6	2.5	2.37-93.5	
TNM stage						
Tumor size	T2	9	15.6±30.5	2.6	0.42-93.5	0.4
	T3	13	27.8±52.3	10.1	0.4-194.9	
	T4	17	20.2±62.2	3.4	0.28-260.8	
Lymph nodes	N0	9	19.07±31.51	2.7	0.4-93.5	0.9
	N1	23	27.24±64.91	4.9	0.28-260.8	
	N2/N3	7	6.70±5.14	5.1	2.5-17.5	
Immunohistochemistry:						
ER	Positive	27	25.3±59.7	4.8	0.28-260.8	0.09
	Negative	12	11.01±14.1	6.4	0.4-43.02	
PR	Positive	30	17.1±37.6	4.1	0.28-194.9	0.6
	Negative	9	42.7±96.3	10.1	0.4-260.8	
HER2neu	Positive	13	8.5±12.3	3.2	0.28-43.02	0.5
	Negative	26	29.03±63.7	4.9	0.4-260.8	
KI67	High	31	26.4±57.6	5.2	0.4-260.8	0.1
	Low	7	3.44±3.84	1.4	0.28-10.8	
Molecular	luminal A	5	2.6±2.2	1.4	0.43-5.27	0.31
subtypes	luminal B	29	27.5±59.5	4.9	0.28-260.8	
	Her2 positive disease	2	10.5±0.5	10.5	10.1-10.83	
	Triple negative disease	3	4.6±5.6	2.4	0.4-10.98	

*, Significant at P ≤ 0.05

**Table 4a:** Correlation between miR-34a & miR-125b Expression Levels and Response to Therapy in BC Patients

MiRNAs	Subgroups	No.	Mean ± SD	Median	Range	P-value
MiRNA-34a	CR+PR	27	1182.6±2849.9	25.9	1.54-11307.6	
	SD+PD	12	2680.2±5417.6	78.3	11.20-17020.7	0.14
MiRNA-125b	CR+PR	27	19.32±40.35	5.23	0.28-194.9	
	SD+PD	12	26.94±74.06	2.85	0.40-260.8	0.4

CR, complete remission; PR, partial remission; SD, stationary disease; PD, progressive disease; *, Significant at P ≤ 0.05

**Table 4b:** Correlation between miR-34a & miR-125b Expression Levels and Response to Therapy in BC Patients

MiRNAs	Subgroups	No.	Mean ± SD	Median	Range	P-value
MiRNA-34a	CR+PR+SD	35	984.9±2537.8	43.6	1.54-11307.6	
	PD	4	7405.4±7867.8	6281.6	37.7-17020.7	0.03*
MiRNA-125b	CR+PR+SD	35	16.5±35.9	4.9	0.28-194.96	
	PD	4	67.1±129.2	3.6	0.4-260.8	0.7

CR, complete remission; PR, partial remission; SD, stationary disease; PD, progressive disease; *, Significant at P ≤ 0.05

**Figure 1:**
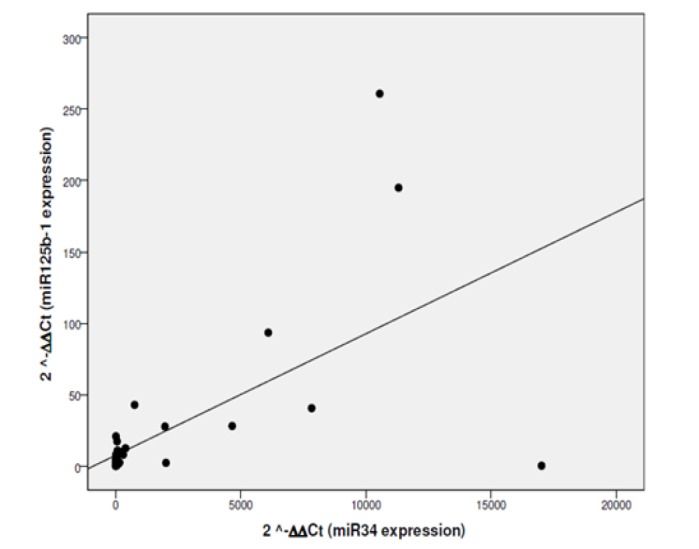
Correlation of miR34 and miR125b Expression Levels in BC Group

*miR-34a expression in controls and breast cancer patients*

In control group, miR-34a expression values ranged between 0.52 and 1.95 with a mean value of 1.13 ± 0.54 and median value of 1.13, while in BC patients, it ranged between 1.54 and 17020.7 with a mean value of 1643.4 ± 3813.6 and median value of 47.3. miR-34a expression levels were significantly higher in BC patients compared to controls with p value <0.001. Correlations of *miR-34a* expression levels with patients` characteristics were described in Table 2. ROC (receiver operating characteristic) curve analysis performed to evaluate the diagnostic value for the miR-34a with AUCs (area under the ROC curves) = 0.995. For distinguishing BC patients from normal controls, ROC curve provides a cutoff point for diagnosis of the disease, below it (2.57) were considered as “Negative”, while those with expression level higher than or equal to 2.57 were considered as “Positive”. We also, found that sensitivity was 97.4%, specificity was 100%, PPV was 100%, NPV was 83.3% and accuracy was 97.7%*.*

*miR-125b expression in controls and breast cancer patients*

In control group, *miR-125b* expression values ranged between 0.7 and 1.53 with a mean value of 1.04 ± 0.35 and median value of 0.92, while in BC patients, it ranged between 0.28 and 260.8 with a mean value of 21.7 ± 52.1 and median value of 4.75. *miR-125b* expression levels were insignificantly higher in BC patients compared to controls with p value = 0.2. Correlations of *miR-125b* expression levels with patients` characteristics were described in Table 3. ROC curve analysis performed to evaluate the diagnostic value for the *miR-125b* with AUCs = 0.68. For distinguishing BC patients from normal controls, ROC curve provides a cutoff point for diagnosis of the disease, below it (8.69) were considered as “Negative”, while those with expression level higher than or equal to 8.69 were considered as “Positive”. We also, found that sensitivity was 66.7%, specificity was 70.0%, PPV was 90.6%, NPV was 41.2% and accuracy was 73.5%.

*Correlation between miR-34a and miR-125b expression levels and response to therapy in locally advanced BC patients*

Twenty seven (69.2%) patients achieved complete response (CR) or partial response (PR), while 12/39 (30.7%) patients still had stationary disease (SD) or progressive disease (PD). Median miR-34a expression levels in BC patient with SD or PD were higher than the levels in patients with CR or PR. However, it doesn’t reach statistically significant difference with P value = 0.14. Only patients with progressive BC disease had significantly higher miR-34a expression levels with p value= 0.03*. Median levels of miRNA-125b expression in BC patient with CR or PR were insignificantly higher than patients with SD or PD with P value = 0.38 (Table 4a and 4b).

*Correlations between miR-34a and miR-125b in BC patients:*

There is direct proprotional relation between *miR-34a *and *miR-125b* expression levels with correlation coefficient (r = 0.58). Statistical analysis shows highly significant statistical correlation between *miR-34a *and *miR-125b* expression levels with P value <0.001* (Figure 1).

## Discussion

Breast cancer is a heterogeneous disease with many etiological risk factors including genetic and environmental factors. The growing awareness of the molecular pathogenesis of cancer is providing new targets for early diagnosis, disease characterization, patients’ risk stratification, development of predictive biomarkers for monitoring disease progress and therapy effectiveness, etc. Heneghan et al., (2011) reported that miRNAs show great potential as diagnostic and prognostic biomarkers for BC. Although the clinical application of serum miRNAs as a noninvasive strategy is promising, the miRNA signatures should be further investigated in BC patients. In the current study, we analyzed the serum level of two miRNAs which are *miR-34a* and* miR-125b* in 39 newly diagnosed locally advanced breast cancer females and 10 age and sex matched healthy volunteers. The *miR-34a *gene is located at lp36.23, it has been identified as a target of* P53* and acted as a tumor suppressor (Misso et al., 2014). Also, Tang et al., (2012) revealed that miR-34a may be involved in regulation of the process of multi-drug resistance (MDR) in BC by targeting *BCL-2*, *CCND1*, and NOTCH1. So,* miR-34a* can serve as an indicator of MDR and prognosis of BC patients. In our study, we found that *miR-34a* expression levels were significantly higher in BC patients compared to controls with p value <0.001. Our result is in agreement with Roth et al., (2010) who reported that *miR-34a* could be used for BC diagnosis because BC patients have higher serum *miR-34a* expression than healthy females, making it as a promising biomarker with another reports revealed an important association between miR-34a and BC risk (OR = 3.12, 95% CI: 1.83–4.39, P < 0.001). While, not in agreement with My (2014) who found lower *miR-34a* levels in advanced BC cell lines and significantly reduced circulating *miR-34a *levels in sera of BC patients with lower *miR-34a* levels in higher stages. These findings may be explained by the difference in sample size and methodical procedures. We performed ROC curve analysis to evaluate the diagnostic value for the *miR-34a* with AUCs = 0.995 and a cutoff point of 2.57. We found that sensitivity was 97.4%, specificity was 100%, PPV was 100%, NPV was 83.3% and accuracy was 97.7%. Our results were similar to Imani et al., (2017) who reported that *miR-34a* had more promising accuracy for BC diagnosis with the AUC of the summary receiver operating characteristic (SROC) was 0.80. Accordingly, *miR-34a* is highly accurate as an independent diagnostic biomarker for BC. We found that *miR-34a* expression levels in non responsive BC patients were insignificantly higher than the levels in responsive patients. Only patients with progressive BC disease had significantly higher *miR-34a *expression levels with p value= 0.03*. However, Li et al., (2017) found that patients with* miR-34a* low expression had poorer OS and DFS compared to those with high expression, suggesting that low *miR-34a* expression indicates poor prognosis for breast cancer patients. In our work, we also investigated *miR 125b* which is a tumor suppressor in breast tumorigenesis. We found that *miR-125b *expression levels were insignificantly higher in BC patients compared to controls. This is contradictory to Mar-Aguilara et al., (2013) who revealed the expression of miR125b was significantly higher in BC sera than in healthy controls. Also, Wang et al., (2012) found that early stage BC patients had similar *miR-125b* level to healthy controls, late stage patients had on average 3.5-fold higher mean values of *miR-125b* than early stage patients and healthy controls with p value < 0.01. However, Han et al., (2013) reported that the serum concentrations of *miR-125b *showed no difference between BC patients and healthy controls. We performed ROC curve analysis to evaluate the diagnostic value for the *miR-125b* with AUCs = 0.68 and a cutoff point of 8.69. We found that sensitivity was 66.7%, specificity was 70.0%, PPV was 90.6%, NPV was 41.2% and accuracy was 73.5%. Also, Mar-Aguilara et al., (2013) found that* miR-125b* had AUC = 0.95 of ROC with the cutoff of 8.46, the sensitivity and specificity were 88.90% and 80.00%, respectively. Our levels of *miRNA-125b *expression in responsive BC patients were insignificantly higher than non responsive patients. However, Wang et al., (2012) reported that miR-125b was associated with therapeutic response exhibiting higher expression level in non-responsive patients which reach statistically significant difference with p = 0.008. 

In conclusion, miRNAs became a rising issue in cancer genetics; they are crucial candidates for novel molecular targeted therapies due to their capability to regulate numerous genes in molecular pathways. Our data suggest that circulating *miR-34a* and* miR-125b* expression levels are promising non-invasive biomarkers in diagnosing BC with direct highly significant correlation between both. *MiR-34a *expression levels were associated with chemotherapeutic resistance as higher levels were found in non-responsive patients. Further studies are needed to evaluate the potential role of these biomarkers as developing therapeutic agents for non responsive patients. 
